# Crystal Structure of the ZrO Phase at Zirconium/Zirconium Oxide Interfaces**

**DOI:** 10.1002/adem.201400133

**Published:** 2014-06-27

**Authors:** Rebecca J Nicholls, Na Ni, Sergio Lozano-Perez, Andrew London, David W McComb, Peter D Nellist, Chris RM Grovenor, Chris J Pickard, Jonathan R Yates

**Affiliations:** Dr. R. J. Nicholls, Dr. S. Lozano-Perez, A. London, Prof. P. D. Nellist, Prof. C. R. M. Grovenor, Dr. J. R. Yates, Department of Materials, University of OxfordParks Road, Oxford, OX1 3PH, UK; Dr. N. Ni, Department of Materials, Imperial College LondonLondon, SW7 2AZ, UK; Prof. D. W. McComb, Materials Science and Engineering, The Ohio State UniversityColumbus, Ohio 43210, USA; Prof. C. J. Pickard, Department of Physics and Astronomy, University College LondonGower Street, London, WC1E 6BT, UK

## Abstract

Zirconium-based alloys are used in water-cooled nuclear reactors for both nuclear fuel cladding and structural components. Under this harsh environment, the main factor limiting the service life of zirconium cladding, and hence fuel burn-up efficiency, is water corrosion. This oxidation process has recently been linked to the presence of a sub-oxide phase with well-defined composition but unknown structure at the metal–oxide interface. In this paper, the combination of first-principles materials modeling and high-resolution electron microscopy is used to identify the structure of this sub-oxide phase, bringing us a step closer to developing strategies to mitigate aqueous oxidation in Zr alloys and prolong the operational lifetime of commercial fuel cladding alloys.

## 1. Introduction

Zirconium-based alloys have been used as nuclear fuel cladding and structural components in water-cooled nuclear reactors since the 1950s. Much effort has gone into optimizing these materials to withstand the harsh service environments and to contribute to the safe operation and long lifetime of these reactors.[1] The main factor limiting the service life of zirconium cladding and hence fuel burn-up efficiency is water corrosion.[1]

The oxidation of zirconium alloys is a complex process, which starts by the alloy developing a black protective oxide resulting in a parabolically decreasing oxidation rate. When the oxide is about 2 μm thick there is an abrupt transition to a faster oxidation rate, and in some samples several of these cycles can be observed in the weight gain kinetics.[2] Because the oxidation mechanism involves the transport of oxidizing species through the growing oxide layer to react with the metal surface, the metal/oxide interface has been a particular focus of experimental observations. Many authors have described a thin “intermediate” or “sub-oxide” layer at this interface, for instance phases with stoichiometry Zr_3_O[3] or ≈40 at% O[4] have been reported. More recently, electron energy loss spectroscopy (EELS)[5] and atom probe tomography[6,7] have identified a layer of composition very close to ZrO at the interface between the metal and the oxide under conditions of slow oxide growth before the first abrupt transition to faster oxidation kinetics. A cubic ZrO phase has been reported at this interface,[8,9] but this identification has been challenged,[10,11] and despite considerable experimental effort, the structure of the ZrO sub-oxide is still unknown. Interestingly, this layer is not found during the fast, post-transition stage of oxidation, and this is one of the most obvious microstructural changes as the rate increases. Even though we cannot be sure if the ZrO layer is responsible for the slower oxidation kinetics, or is stabilized only when the metal/oxide interface is moving slowly, knowing the structure of this phase is critical to developing a full understanding of the mechanism of oxidation.

There is no known stable bulk form of ZrO. A cubic rocksalt structure[12–14] has previously been suggested to exist, however, it has not been possible to find an unambiguous experimental report of this structure in the literature (see Supporting Information). Very recently, Puchala and Van der Ven[15] have used a cluster expansion approach to predict a stable hexagonal ZrO phase based on the δ-TiO structure. Prediction of new, unknown, crystal structures has long been a challenge for theoreticians. In recent years, approaches using numerical techniques such as genetic algorithms,[16–20] particle swarm,[21] and random structure generation[22] have been shown to be capable of predicting new phases of materials. There has been particular success identifying high-pressure phases of simple materials (e.g., [23]), with several ab initio predictions subsequently being confirmed experimentally.

To identify the structure of the sub-oxide material, we propose to first obtain the lowest energy structures of ZrO using modeling techniques based on density functional theory (DFT). The growth of the layer will be influenced by its interface to the metal and we therefore do not expect the sub-oxide layer observed experimentally to necessarily be the structure with the lowest formation energy, although it should be among the set of structures with the lowest energies. If a low energy structure of ZrO is indeed the form which occurs at the Zr/ZrO_2_ interface, then its predicted properties must also match those observed. In this case, the experimental data available for comparison includes electron diffraction, a direct probe of the atomic arrangement, and electron-energy loss experiments, which are a sensitive probe of the electronic structure of the material.

## 2. Candidate ZrO Structures

**Fig 1:**
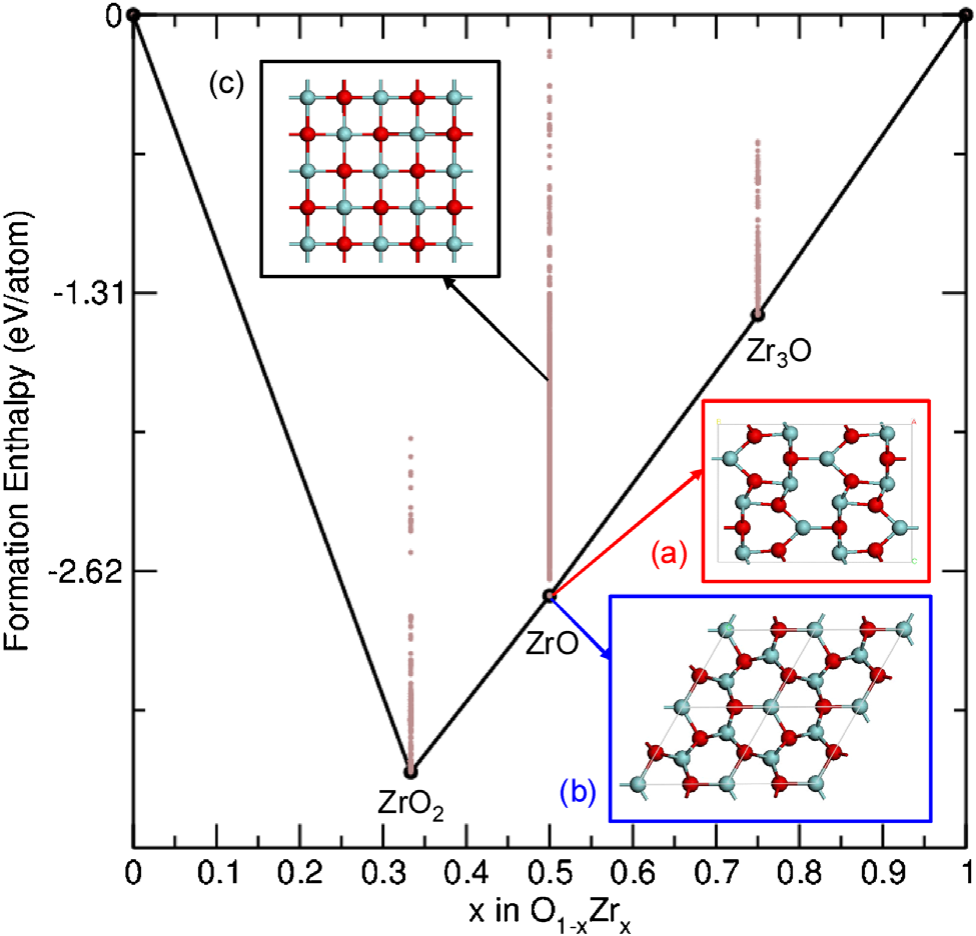
Convex hull of the O_(1−*x*)_Zr*_x_* system. The two stable forms of ZrO are shown; the orthorhombic structure (a) has *Cmcm* symmetry (*a* = 3.24 Å, *b* = 12.21 Å, and *c* = 8.59 Å) and the hexagonal structure (b) has *P-62m* symmetry (*a* = 5.31 Å and *c* = 3.20 Å). The experimentally reported cubic rock salt structure (c) is also shown (*a* = 4.62 Å). Oxygen atoms are shown in red.

We generated candidate ZrO structures using an ab initio random structure searching (AIRSS) algorithm.[22,24] Figure 1 shows the relative energies of the predicted ZrO structures together with a number of other Zr*_x_*O*_Y_* stoichiometries. The search identified two low energy ZrO structures, which are energetically stable with respect to decomposition into ZrO_2_ and Zr_3_O. The first structure is orthorhombic (Figure 1a), the second is hexagonal (Figure 1b) and iso-structural with ε-TaN.[25] This hexagonal structure is the same as that recently predicted by Puchala and Van der Ven[15] on the basis of its similarity to phases in the TiO system. Despite being structurally rather different, the orthorhombic and hexagonal structures have predicted formation energies within 0.002 eV per formula unit of each other. Cubic ZrO was not found by the AIRSS algorithm, which is consistent with previous calculations, which reported it to be mechanically unstable as a bulk phase.[11] We find its formation energy to be 1 eV per formula unit higher than the orthorhombic and hexagonal phases. Thus, we consider both the orthorhombic and hexagonal structures to be candidate structures for the ZrO phase and we also include the cubic structure for consideration due to its previous prominence in the literature. We now evaluate the candidate structures by comparing their predicted properties with experiment.

## 3. Comparison of Candidate Structures and Experimental Data

**Fig 2:**
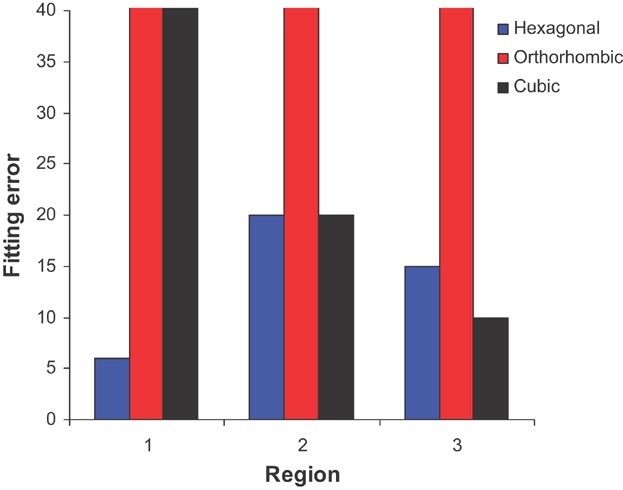
Fitting error of simulated diffraction data from each of the three different structures with experimental data from three different interface regions.

We have selected five convergent beam electron diffraction patterns from the interface region (three of which are shown in Figure S1 and S2 of the Supporting Information). Using a crystallography software package,[26] the fit with the experimental data was evaluated for each of the candidate structures by summing the relative errors in both interplanar distances and angles (see Supporting Information). Two of the experimental patterns were not matched by any of the candidate structures. We note that isolating the pattern from a single sub-oxide grain is difficult as the grain diameters are usually smaller than the sample thickness so it is possible that these patterns have contributions from more than one grain (and/or from the adjacent oxide or matrix). The fit between the other three experimental diffraction patterns and the proposed structures is summarized in Figure 2. In cases where a particular experimental pattern could not be matched by a candidate structure, an arbitrarily large error has been included. The hexagonal structure is consistent with all three diffraction patterns, the cubic structure with two while the orthorhombic structure is not consistent with any of the experimental data.

**Fig 3:**
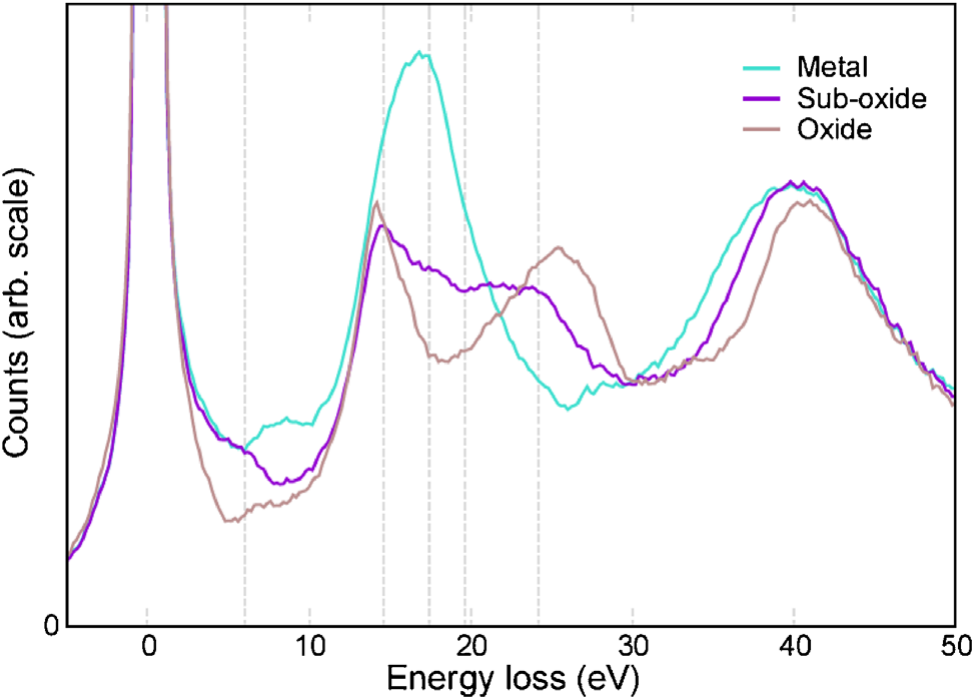
Experimental low-loss EELS spectra from the metal, sub-oxide, and oxide.

**Fig 4:**
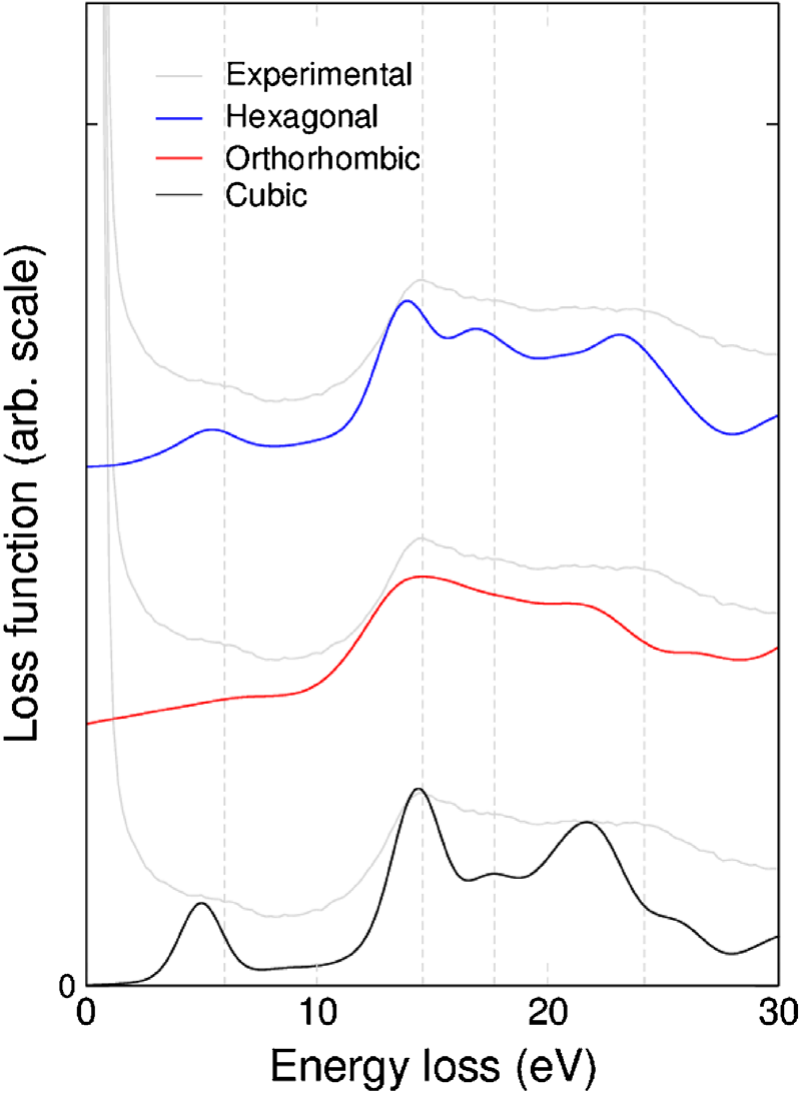
Comparison of simulated EELS spectra from each of the three candidate ZrO structures with experimental data.

We also obtained a series of low-loss EELS spectra across the interface region. The spectra can be seen to change from that of Zr metal on one side of the interface to the characteristic ZrO_2_ spectrum on the other. In between there is a region in which the spectrum is not a linear combination of the other two. Examples of these three spectra are given in Figure 3 and in the Supporting Information (Figure S3). The peak positions for the different spectra are summarized in Table 1. EELS spectra have been simulated for each of the candidate structures using DFT and the random phase approximation excluding local field effects (RPA-NLF) (Figure 4). We focus on the first 30 eV of the spectra as this is the most diagnostic region. Higher level calculations have shown the RPA-NLF approach to be reliable in this energy regime.[27] The positions of the peaks simulated from the three different structures are listed in Table 1, along with a simple estimate of their match to the experimental data from the suboxide region. The comparison of experimental and computed spectra is complicated by the fact that the spectra from the hexagonal and orthorhombic structures depend on the orientation of the crystal with respect to the electron beam. However, this anisotropy is very small for the orthorhombic case and mainly affects the peak intensities in the hexagonal case (see Supporting Information). We note that small variations are seen in the experimental data recorded from the interface region both in different parts of a sample and in different samples (Supporting Information Figure S4). This may indicate a small orientation dependence in the spectra as the data may correspond either to grains in different orientations or from multiple grains.

**Table 1:** Peak positions for the experimental EELS spectra collected from the metal, sub-oxide, and oxide along with the peak positions from the data simulated from the three model sub-oxide structures

Experiment					

	Peak positions [eV]				
Metal	7.4	16.8			
Sub-oxide	6.0	14.6	17.4		
Oxide	6.8	14.4	25.4	24.2	

Simulation					

	Peak positions [eV]				Comparison with experiment
Hexagonal	5.5	13.9	16.9	23.1	1.5
Orthorhombic	6.2	14.4	17.0	22.1	2.2
Cubic	5.0	14.4	17.7	21.7	2.7

The error in the measurement of the experimental peak positions is ±0.2 eV. An estimate of match between experiment and simulation for the sub-oxide has been calculated using 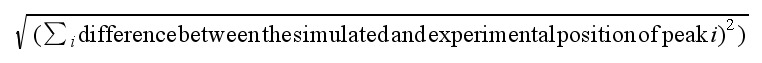.

When comparing peak positions, the spectrum simulated from the hexagonal structure is found to have the closest match to experiment. This conclusion does not change if the orientation dependence is also taken into account (see Supporting Information Table S1). The intensities of the peaks are also significant, but these are more difficult to compare with experiment due to the presence of the zero-loss peak (at 0 eV) in the experimental data. The three peaks at 14.6, 17.4, and 24.2 eV are all of a similar height in the experimental data. These features are well matched by the data simulated from the hexagonal and orthorhombic structures, but less well matched the cubic structure where the spectrum shows a reduction in intensity for the second of the three peaks.

The oxygen K-edge was also obtained experimentally across the interface region, although the lack of strong features make it less diagnostic than the low-loss regime (see Supporting Information). Simulated spectra from the three candidate structures can be seen in the Supporting Information (Figure S8) and both the hexagonal and orthorhombic structures are consistent with the experimental data. The zirconium L_2,3_-edge obtained from the interface region showed a systematic downwards shift in energy from the oxide to the metal consistent with a decrease in oxidation number (see Supporting Information Figure S10).

In summary, the hexagonal structure is the only candidate ZrO structure, which is both calculated to be stable and able to explain data from both diffraction and EELS experiments. We therefore propose that the sub-oxide phase has the hexagonal structure shown in Figure 1b. The bandstructure and density of states of the structure are shown in the Supporting Information. The AIRSS algorithm produced two energetically equivalent structures, so why might the hexagonal phase be favored over the orthorhombic? The sub-oxide grains form at the boundary between the ZrO_2_ and the Zr metal by transport of the oxidizing species through the oxide and into the metal. The Zr metal also has a hexagonal structure, and the metal immediately below the sub-oxide contains a significant concentration of oxygen in solution, increasing the lattice parameters of the metal from *a* = 3.236 Å, *c* = 5.150 Å to *a* = 3.253 Å, *c* = 5.186 Å.[28] The predicted lattice parameters of the hexagonal ZrO phase (*a* = 5.31 Å, *c* = 3.20 Å) are such that oxygen-saturated metal and the ZrO grains can fit together with only ≈2% mismatch at the boundary. We suggest that the crystallographic similarity between the Zr metal and hexagonal ZrO structures may be the reason this phase forms in preference to the orthorhombic phase.

## 4. Conclusion

We have determined the structure of the ZrO sub-oxide phase found at the zirconium metal/oxide interface by using a combination of theoretical structure predication and experimental techniques. Knowing both the structure and chemistry of the ZrO phase at the metal/oxide interface during waterside corrosion of Zr alloys in nuclear fuel assemblies now allows us to model how changes in alloy chemistry may be used to control the corrosion susceptibility in service. Importantly, neither experimental or theory alone were able to solve the problem. This work not only provides an answer to an important technological question, but also showcases the power of this combination of techniques.

## 5. Experimental

### 5.1. Sample Preparation

Two commercial zirconium alloys (Zircaloy-4 and ZIRLO) currently used for nuclear fuel cladding were provided by Westinghouse. Recrystallized samples were corrosion tested by EDF in a static autoclave (360 °C and 18 MPa) using simulated primary water chemistry (pure H_2_O with 2 ppm LiOH and 1000 ppm boric acid, de-aeration) to imitate the water environment inside a pressurized water nuclear reactor. To study the progression of the oxidation, samples spent varying amounts of time inside the autoclave. This study focuses on two Zircaloy-4 samples, which have spent 34 and 90 days in the autoclave and two ZIRLO samples, which have spent 34 and 100 days in the autoclave.[6]

Thin foil samples for transmission electron microscopy (TEM) analysis have been prepared using the focused ion beam (FIB) in situ lift-out technique on a FEI FIB 200 instrument and final stage thinning with a Zeiss NVision 40 dual-beam FIB. All of the zirconium alloy samples showed phases at the interface that could not be identified solely by electron diffraction, and previous quantitative analytical EELS work has shown that the composition of this phase always has a Zr:O ratio of 1:1.[5] The EELS data shown in Figures 3 and 4 and in the Supporting Information was obtained from a ZIRLO sample, which had been oxidized for 100 days.

### 5.2. Electron Microscopy

Convergent beam electron diffraction patterns were obtained from regions at the interface using a Philips CM20 TEM operated at 200 kV. The convergent beam is used to limit the volume of the specimen from which diffraction information is obtained and this produces a diffraction pattern in the form of disks rather than sharp spots.

1D spectrum images of the low-loss region were obtained from the metal–oxide interface region using an aberration-corrected Nion UltraSTEM 100 operated at 100 kV and equipped with a Gatan Enfina spectrometer. The energy resolution was 0.6 eV measured (at a dispersion of 0.2 eV per pixel) using the full width half-maximum of the zero loss peak. The spectra were collected in 0.25 nm steps and 50 0.04 s exposures were combined for each pixel to give a total integration time of 2 s.

Spectrum images were also obtained using an FEI Titan 80–300 fitted with a Gatan Tridiem spectrometer. The full width half-maximum of the zero loss peak was 1.1 eV or less. 0.01 s exposures were used for the collection of low-loss spectra, 10 s exposures for oxygen K-edge and 8 s exposures were used to collect the zirconium L_2,3_-edge data.

### 5.3. AIRSS and Geometry Optimization of Candidate Structures

All DFT calculations have been performed with the CASTEP code[29] using ultrasoft pseudopotentials and the PBE exchange-correlation functional. The AIRSS runs have been performed at 0 GPa for unit cells containing up to six ZrO formula units, and a variety of other compositions. Symmetry constraints were used, with space groups chosen randomly. Three thousand thirty-two structures were generated in total, with 1162 for the ZrO composition. Relatively coarse convergence parameters were employed during the searches, with a cut-off energy of 340 eV, and a k-point sampling of 2*π* × 0.1 Å^−1^ or less.

The final geometry optimizations were carried out with a cut-off energy of 490 eV, a k-point sampling of 2*π* × 0.03 Å^−1^ or less and the structures were optimized until the forces on each atom did not exceed 1 × 10^−3^ eV Å^−1^. The numerical parameters were carefully checked so that their value did not influence the result of the optimization.

### 5.4. Simulations of Experimental Data

Diffraction patterns were simulated from the DFT optimized structures using CaRIne Crystallography 3.1, which employs the structure factor method.

EELS spectra were calculated with OptaDOS,[30] together with the CASTEP DFT code. The parameters used for the EELS calculations were the same as for the geometry optimizations, except that the k-point sampling was increased until any further increases had no effect on the simulated spectrum. A Gaussian broadening of 2.0 eV was applied to the simulated loss function for direct comparison with the experimental data. The core hole was included in the oxygen K-edge simulations by constructing an oxygen pseudopotential with an electron removed form the 1 s orbital. A supercell was used to separate the periodic images of the core hole so that interactions between them did not affect the simulated spectra. The theoretical spectra were broadened using a Gaussian to represent the energy spread of the electron beam, a fixed Lorentzian to represent the broadening due to the finite life-time of the initial state and an energy dependent Lorentzian to represent the broadening due to finite life-time of the final state.

## Supporting Information

As a service to our authors and readers, this journal provides supporting information supplied by the authors. Such materials are peer reviewed and may be re-organized for online delivery, but are not copy-edited or typeset. Technical support issues arising from supporting information (other than missing files) should be addressed to the authors.

**Fig. S1.** Image showing the region the diffraction pattern was obtained from (a), the corresponding convergent beam electron diffraction pattern (b) and the simulated diffraction pattern from the hexagonal structure (c). Environmental applications of organoclays for sorption of phenol and its derivatives. **Figure S2.** (a and c) Images indicating where the convergent beam diffraction pattern was obtained from and (b and d) the corresponding diffraction patterns. **Figure S3.** HAADF image (a) of the metal-oxide interface and a spectrum image (b) taken along the line marked in (a). EELS spectra (c) from the three different regions marked in the image and on the spectrum image. **Figure S4.** Low-loss EELS spectra obtained from the sub-oxide phase. The data from region 1 comes from one sample and from regions 2 and 3 from different areas of the metal-oxide interface in a second sample. **Figure S5.** Orientation dependence of the simulated EELS spectrum for the hexagonal structure showing the in-plane component (100) and the out-of-plane component (001). **Figure S6.** Orientation dependence of the orthorhombic loss function showing the three independent components (100, 010 and 001). **Figure S7.**HAADFimage (a) of the metal-oxide interface and a spectrum image (b) taken along the line marked in (a). Oxygen K-edge EELS spectra (c) from the three different regions marked in the image and on the spectrum image. **Figure S8.** Simulated oxygen K-edges from the three candidate structures along with experimental data obtained from the sub-oxide region. **Figure S9.** Orientation dependence of the simulated oxygen K-edge spectra from the hexagonal (a) and orthorhombic (b) structures. **Figure S10.** Zr L_2,3_-edges obtained from two different sub-oxide regions. **Figure S11.** Bandstructure of the hexagonal ZrO structure with the Fermi energy marked by a dashed line. **Figure S12.** The Fermi energy is marked by a dashed line. **Table S1.** Comparison of the orientation dependent peak positions from the candidate structures with experimental data.
