# Supplementary material for: Crystal Structure of the ZrO Phase at Zirconium/Zirconium Oxide Interfaces
Source: Adv Eng Mater. 2014 Jun 27;17(2):211–5. doi: 10.1002/adem.201400133 (PMC4393322; doi:10.1002/adem.201400133)
Supplement: Supplementary file 1 [file adem0017-0211-sd1.doc]

Supporting Information

Crystal Structure of the ZrO Phase at Zirconium / Zirconium Oxide Interfaces

Rebecca J. Nicholls*, Na Ni, Sergio Lozano-Perez, Andrew London, David W. McComb, Peter D. Nellist, Chris R. M. Grovenor, Chris J. Pickard, Jonathan R. Yates

**Previous experimental identification of the cubic ZrO phase**

Using diffraction data to unambiguously identify the cubic ZrO phase is very difficult because other potential phases can give very similar diffraction data. Furuta and Motohashi^[S1]^ identified a cubic ZrO phase in an oxidised Zircaloy-2 sample, but the formation of hydrides in their sample cannot be excluded. Moseley and Hudson^[S2]^ also identified the cubic ZrO phase in work where they ruled out the existence of a hydride based on diffraction data from the (200) planes. However, there is only ~0.0005 nm difference in the plane spacings between their cubic ZrO phase and the δ-hydride. Recent work by Sethi *et al.*^[S3]^ also reported the presence of this cubic phase in zirconium oxide thin films based on X-ray powder diffraction patterns, but such patterns would also be explained by the hexagonal phase described in this work, as there are very similar inter-planar spacings in both structures.

[S1] T. Furuta, H. Motohashi, *Journal of Nuclear Materials* **1980**, 95, 303.

[S2] P. T. Moseley, B. Hudson, *Journal of Nuclear Materials* **1981**, 99, 340.

[S3] G. Sethi, P. Sunal, M. W. Horn, M. T. Lanagan, *J. Vac. Sci. Technol. A* **2009**, 27, 577.

**Diffraction patterns obtained from the interface region**

Five experimental convergent beam electron diffraction patterns were obtained from interface regions in a ZIRLO sample which had been oxidised for 100 days. The fit between the experimental diffraction patterns and candidate structures was evaluated by a crystallography software package:

$$\begin{matrix} \text{Fitting error (\%) }=abs\left(\frac{\text{experimental interplanar spacing 1 - predicted spacing 1}}{\text{experimental interplanar spacing 1}}\text{x}100\right) \\ +abs\left(\frac{\text{experimental interplanar spacing 2 - predicted spacing 2}}{\text{experimental interplanar spacing 2}}\mathrm{x}100\right) \\ +abs\left(\frac{\text{experimental angle between planes 1 and 2 - predicted angle}}{\text{experimental angle between planes 1 and 2}}\text{x}100\right) \end{matrix}$$

Three of the patterns (Figures S1b, S2b and S2d) were compatible with the candidate structures.

**(b)**

**(c)**

000

130000

130201130000

131201130000

201130000

131000

332131000

201131000

**ZrO_2_ oxide**

**Zr metal**

**(a)**

**50nm**

**Figure S1.** Image showing the region the diffraction pattern was obtained from (a), the corresponding convergent beam electron diffraction pattern (b) and the simulated diffraction pattern from the hexagonal structure (c).


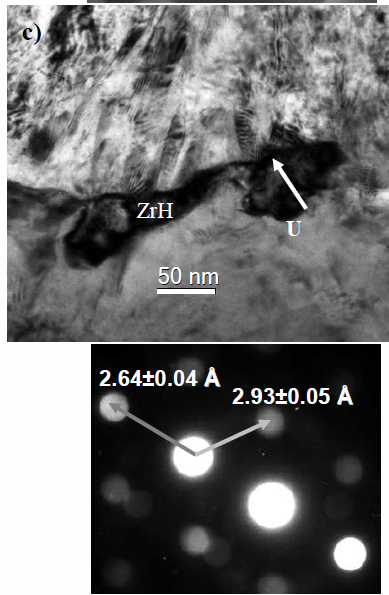


**(a)**

**50nm**

**(b)**

**ZrO_2_ oxide**

**Zr metal**


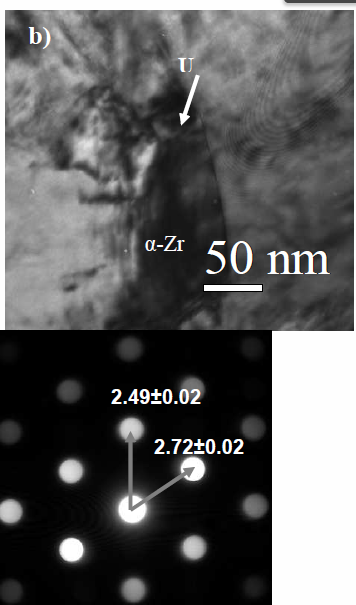


**(c)**

**50nm**

**(d)**

**ZrO_2_**

**oxide**

**Zr metal**

**Figure S2.**  (a and c) Images indicating where the convergent beam diffraction pattern was obtained from and (b and d) the corresponding diffraction patterns.

**Low-loss** **EELS spectra otained from the interface region**

All EELS spectra have been obtained from ZIRLO samples oxidised for 100 days.


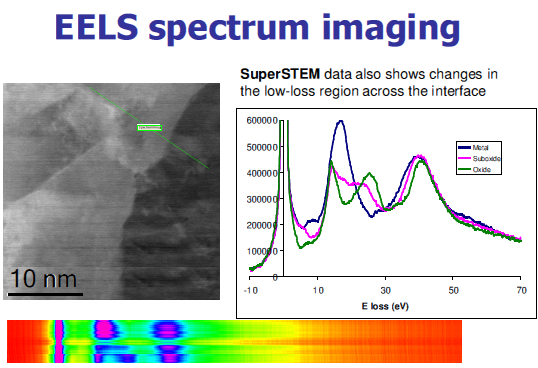

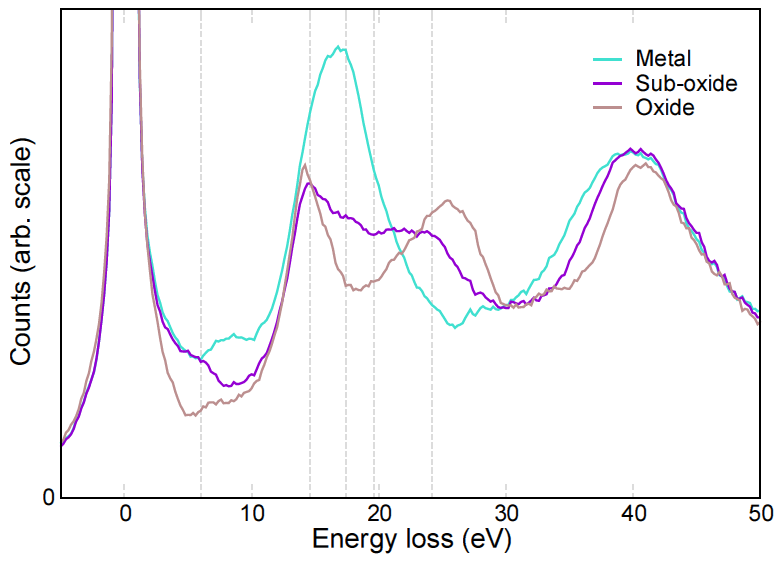


**1**

**2**

**3**


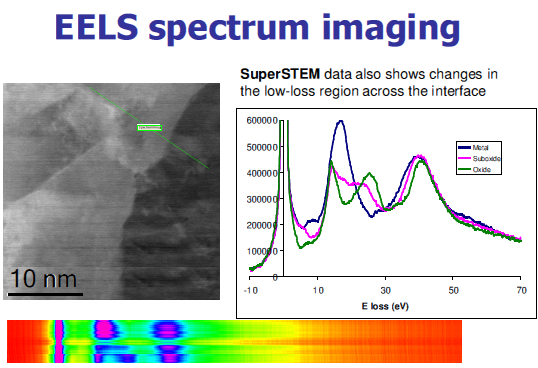


**1**

**2**

**3**

**(a)**

**(c)**

**(b)**

**Figure S3.** HAADF image (a) of the metal-oxide interface and a spectrum image (b) taken along the line marked in (a). EELS spectra (c) from the three different regions marked in the image and on the spectrum image


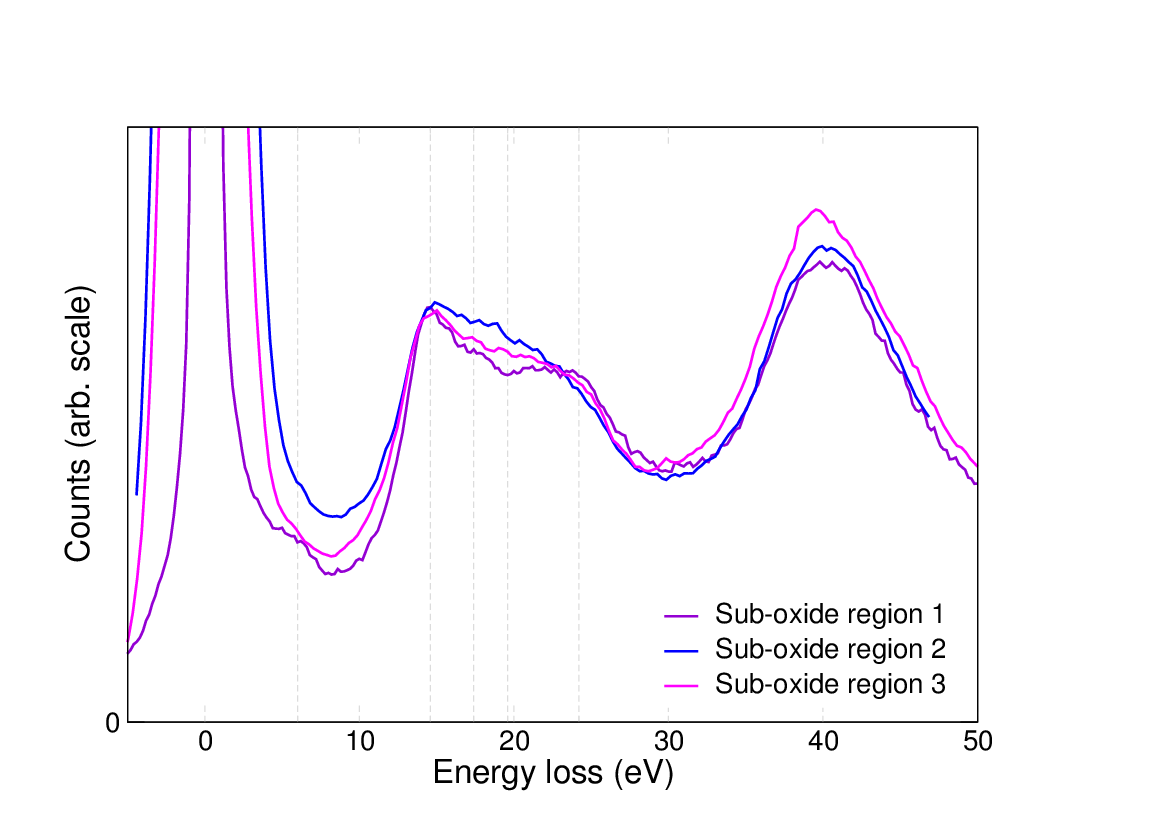


**Figure S4.** Low-loss EELS spectra obtained from the sub-oxide phase. The data from region 1 comes from one sample and from regions 2 and 3 from different areas of the metal-oxide interface in a second sample.

**Orientation dependence of loss function**

The simulated EELS spectrum is related to the dielectric function, which is a tensor. In cubic materials, there is only one independent component in the dielectric tensor, making the EELS spectrum anisotropic. For hexagonal and orthorhombic materials there are two and three independent components respectively, meaning that the EELS spectrum will have an orientation dependence. The orientation dependence of the loss functions simulated from the candidate hexagonal and orthorhombic structures are shown in Figures S5 and S6. Orientation dependence adds an extra complication to the comparison of the simulated data with experiment as it can cause a change in both peak positions and intensities. In terms of the peak positions, the comparison with experiment does not change significantly if orientation is taken into account (Table S1) as none of the components for the orthorhombic structure are a better fit than those for the hexagonal structure. The ratio of the peak heights does not change significantly between components for the orthorhombic structure, but for the hexagonal structure the ratio of the peak heights can be adjusted by changing relative amounts of the two components. As the experimental ratio of the peaks has not been carefully measured (as the zero-loss peak has not been subtracted), we have not attempted to tune the spectrum to the experimental data here.


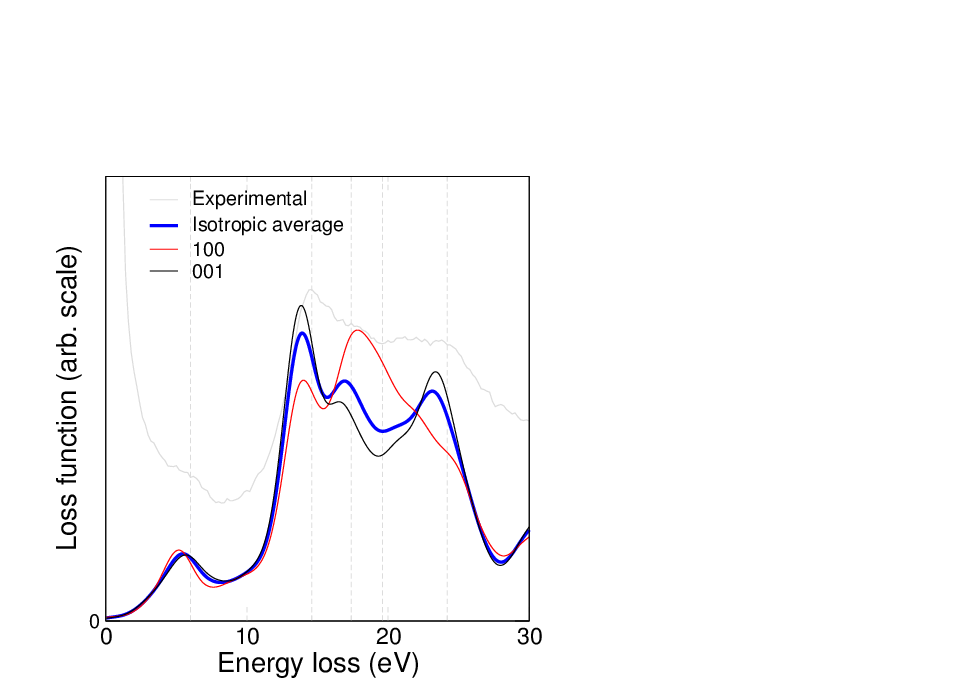


**Figure S5.** Orientation dependence of the simulated EELS spectrum for the hexagonal structure showing the in-plane component (100) and the out-of-plane component (001).


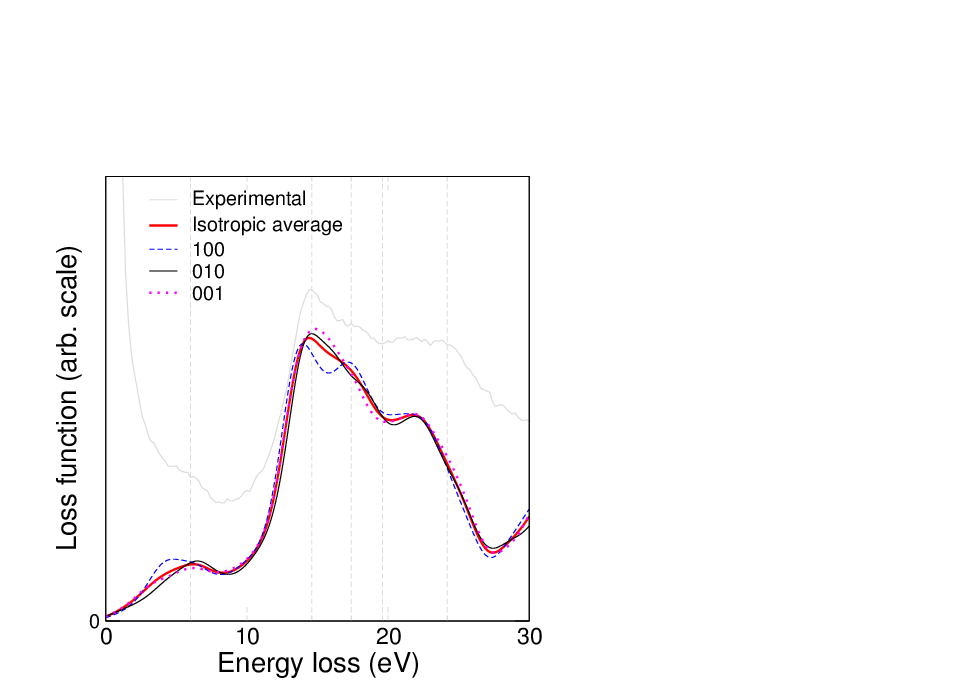


**Figure S6.** Orientation dependence of the orthorhombic loss function showing the three independent components (100, 010 and 001).

| **Experiment** | | | | | | |
| --- | --- | --- | --- | --- | --- | --- |
|  | | **Peak positions (eV)** | | | |  |
| Metal | | 7.4 | 16.8 |  |  |  |
| **Sub-oxide** | | **6.0** | **14.6** | **17.4** | **24.2** |  |
| Oxide | | 6.8 | 14.4 | 25.4 |  |  |
| **Simulation** | | | | | | |
|  | | **Peak positions (eV)** | | | | **Comparison with experiment** |
| **Hexagonal** | **Average** | **5.5** | **13.9** | **16.9** | **23.1** | **1.5** |
|  | 100 | 5.2 | 13.9 | 17.8 | - | 1.1 |
|  | 001 | 5.7 | 13.9 | 16.5 | 23.4 | 1.4 |
| **Orthorhombic** | **Average** | **6.2** | **14.4** | **17.0** | **22.1** | **2.2** |
|  | 100 | 4.8 | 14.0 | 17.1 | 22 | 2.6 |
|  | 010 | 6.5 | 14.6 | 18.5 | 22 | 2.5 |
|  | 001 | 6.2 | 14.8 | 17.5 | 22.1 | 2.1 |
| **Cubic** | | **5.0** | **14.4** | **17.7** | **21.7** | **2.7** |

**Table S1.** Comparison of the orientation dependent peak positions from the candidate structures with experimental data.

**Oxygen K-edge EELS spectra**

**
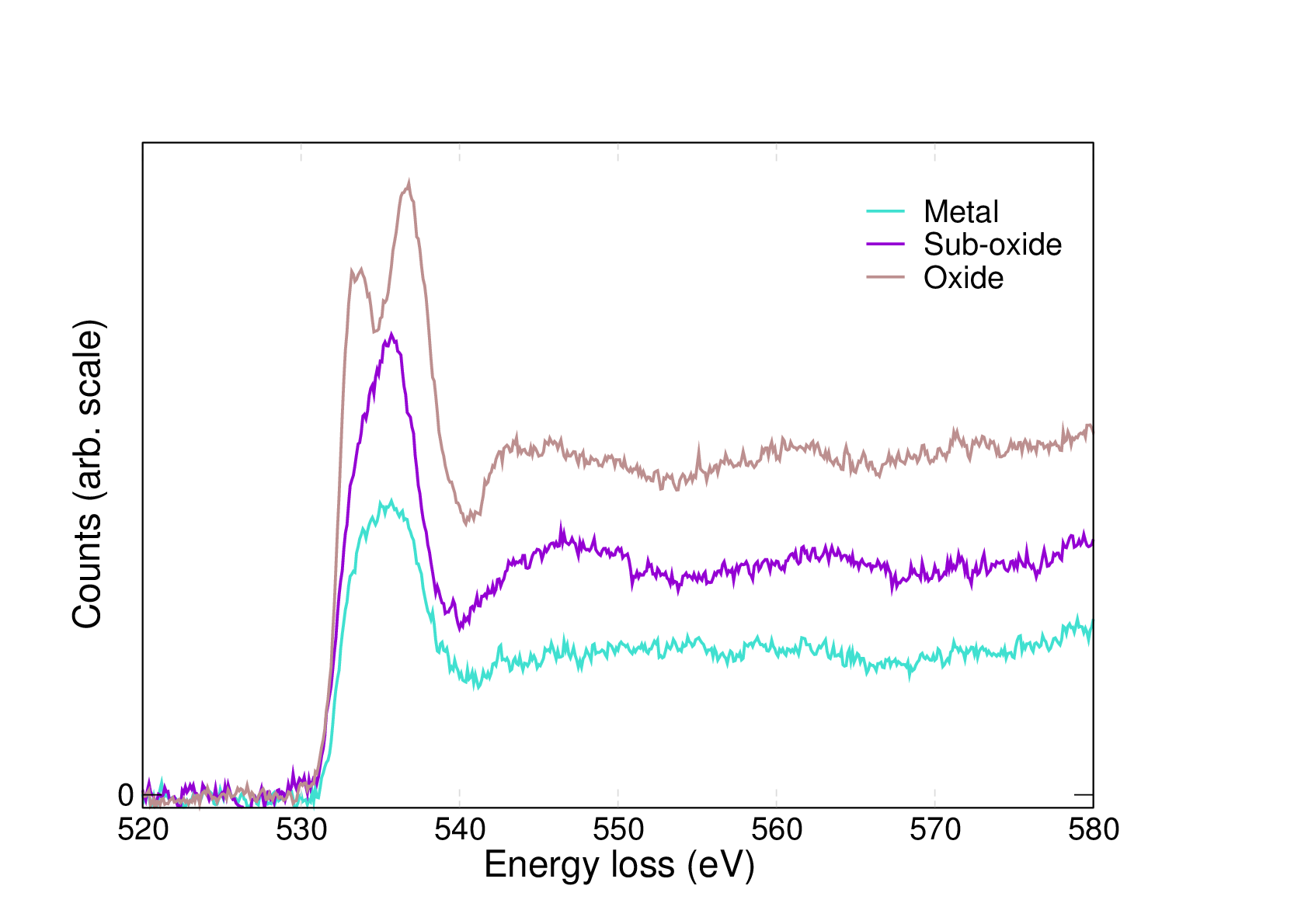
**

**200nm**

**1**

**2**

**3**

**(a)**

**(c)**

**(b)**

**1**

**2**

**3**

**Figure S7.** HAADF image (a) of the metal-oxide interface and a spectrum image (b) taken along the line marked in (a). Oxygen K-edge EELS spectra (c) from the three different regions marked in the image and on the spectrum image.

**
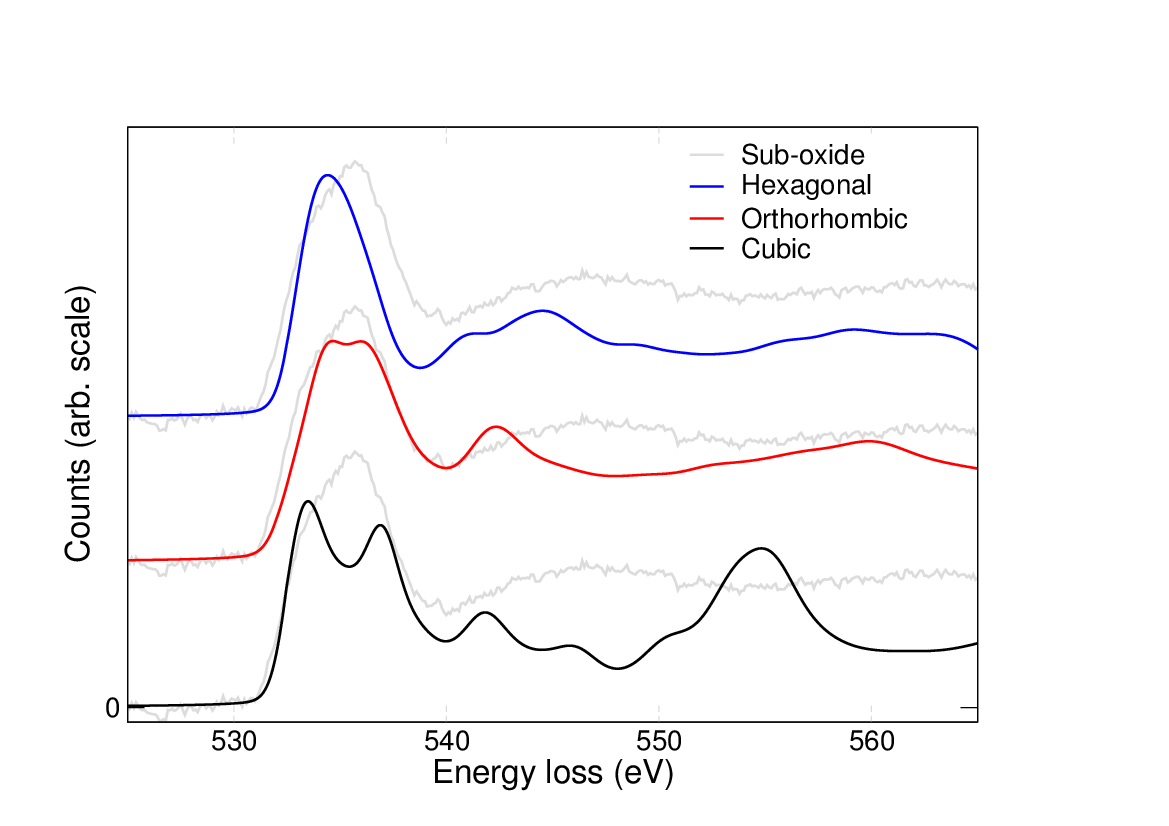
**

**Figure S8.** Simulated oxygen K-edges from the three candidate structures along with experimental data obtained from the sub-oxide region.

(a)

**
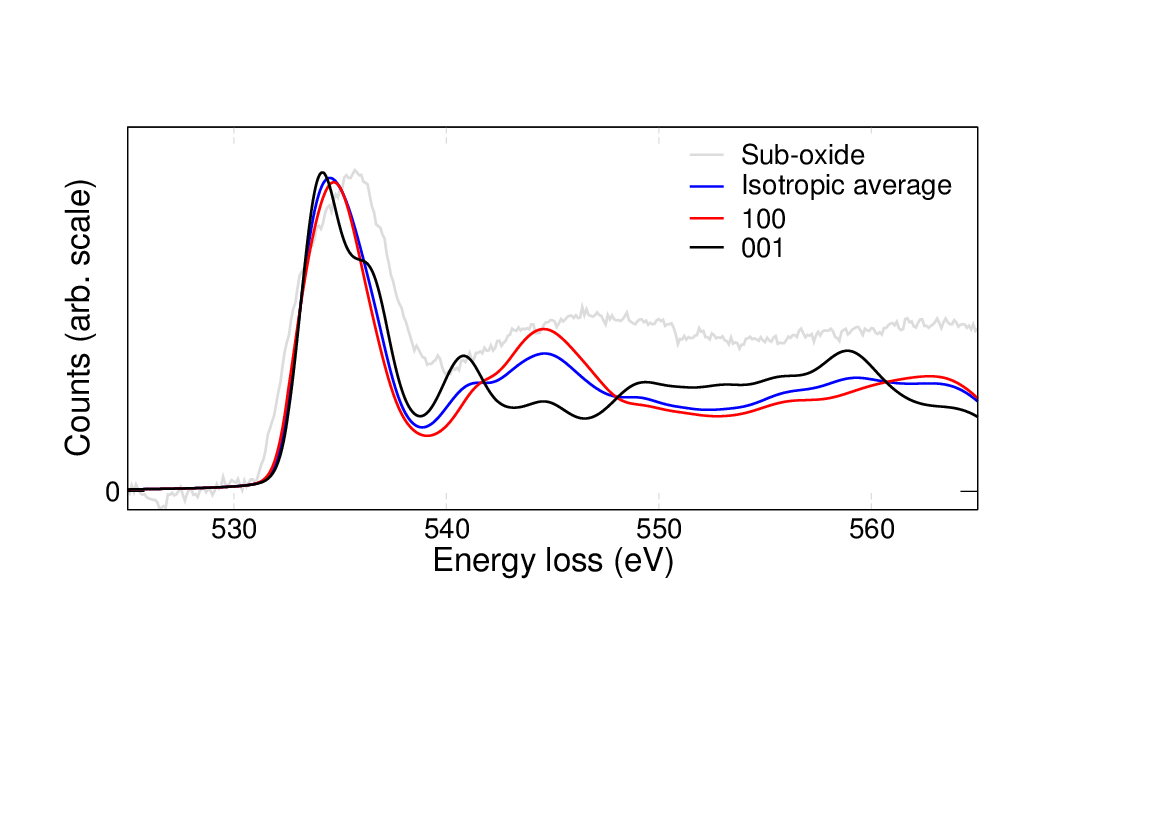
**

**
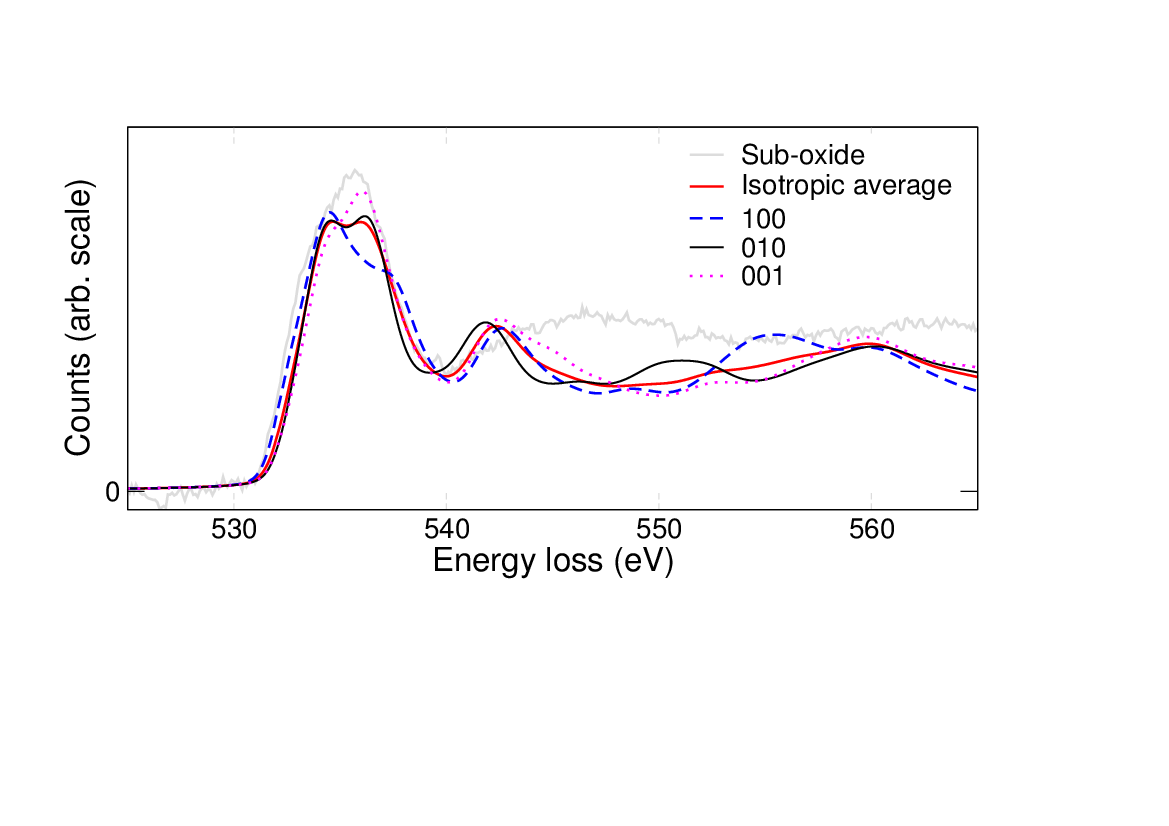
**

(b)

**Figure S9.** Orientation dependence of the simulated oxygen K-edge spectra from the hexagonal (a) and orthorhombic (b) structures

**Zirconium L_2,3_-edge EELS spectra**

**
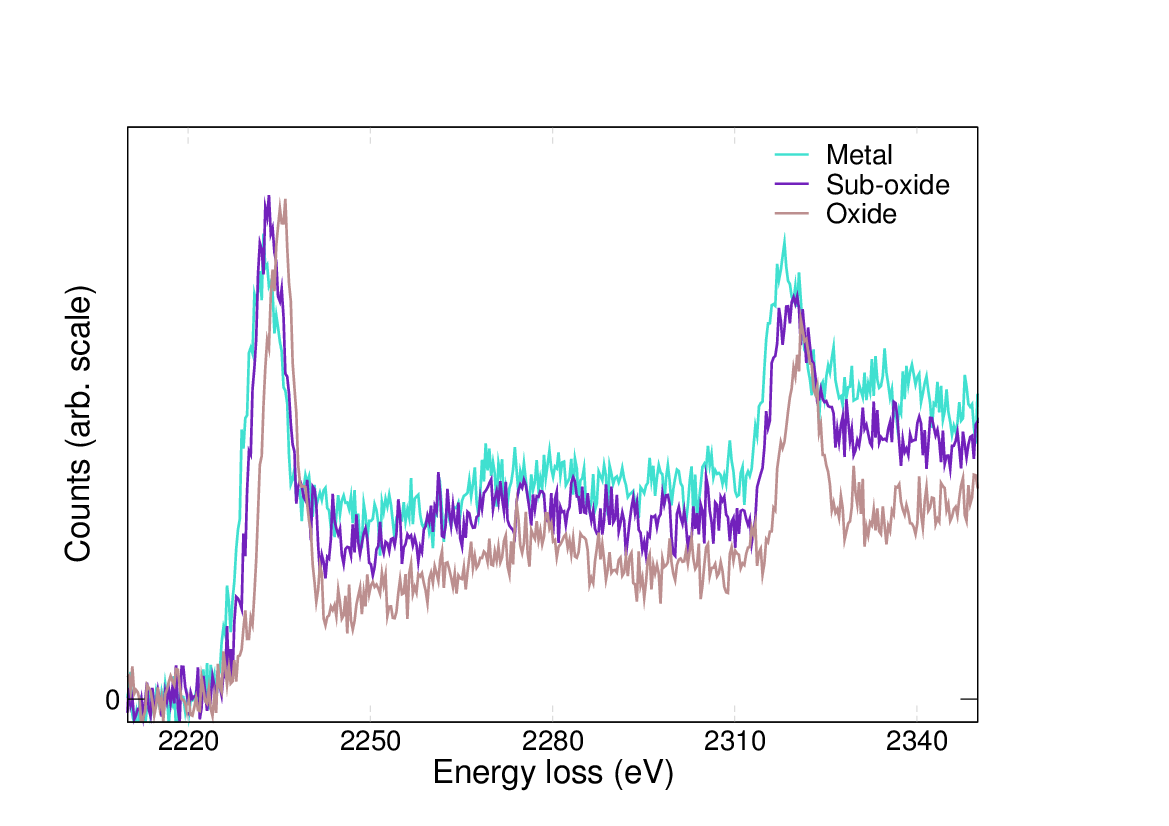

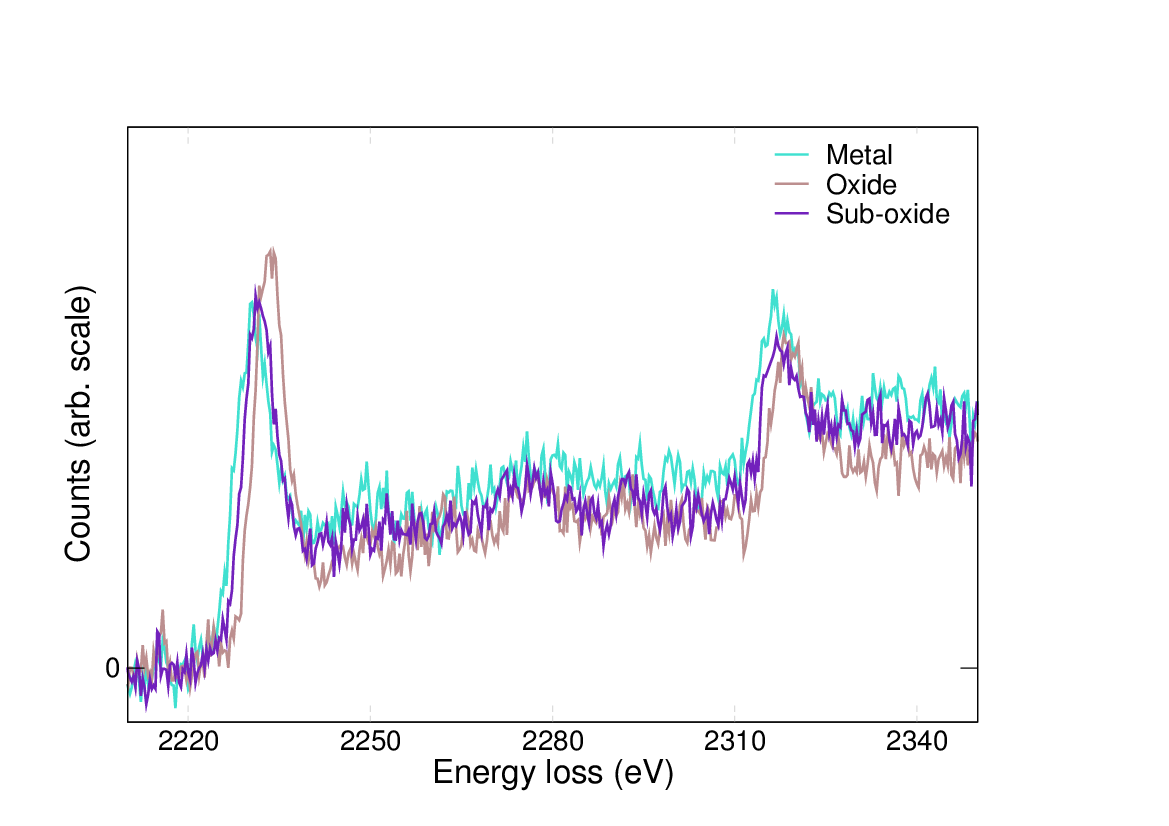
**

**Figure S10.** Zr L_2,3_-edges obtained from two different sub-oxide regions.

There is a shift in the Zr L_2,3_-edge obtained from the metal, sub-oxide and oxide. As the oxidation number decreases from +4 (oxide) to +2 (sub-oxide) to 0 (metal) the edge moves systematically downwards in energy.

**Hexagonal ZrO bandstructure and DOS**

The bandstructure and density of states (DOS) of the of the hexagonal ZrO structure are shown in Figures S6 and S7 respectively. The site projected partial DOS (Figure S7) shows that the states around -37 eV are zirconium s-states, those at about -16 eV are predominantly zirconium p-states and those at -8 eV are mainly oxygen s-states. The slightly more dispersive states at ~5 eV have mainly oxygen p-like character and the states around the Fermi level have mainly zirconium d-like character with small contributions from zirconium p-like and oxygen p-like states.


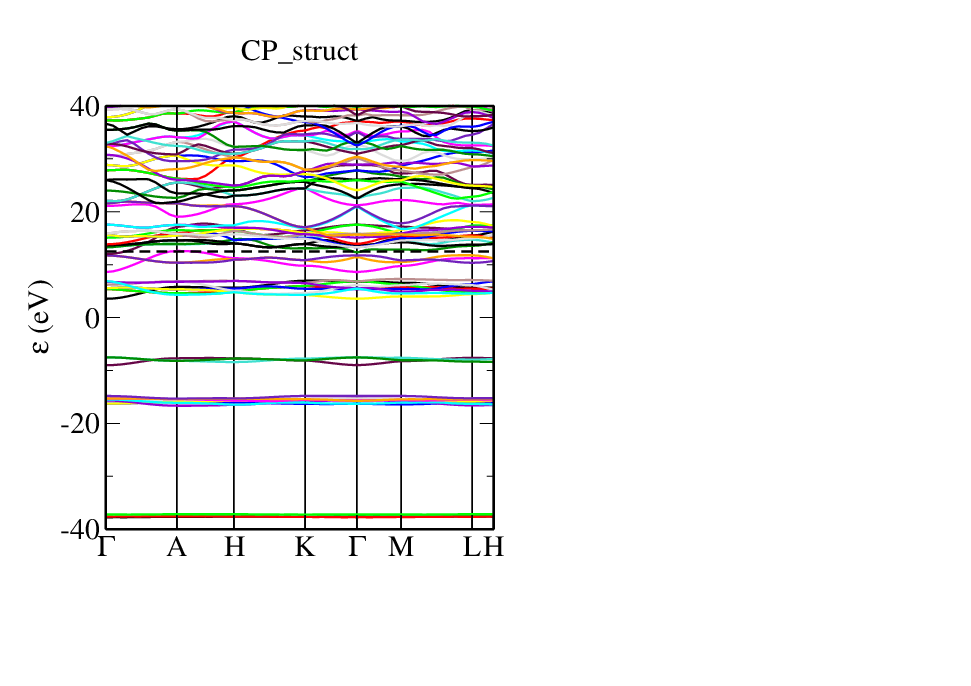


**Figure S11.** Bandstructure of the hexagonal ZrO structure with the Fermi energy marked by a dashed line.


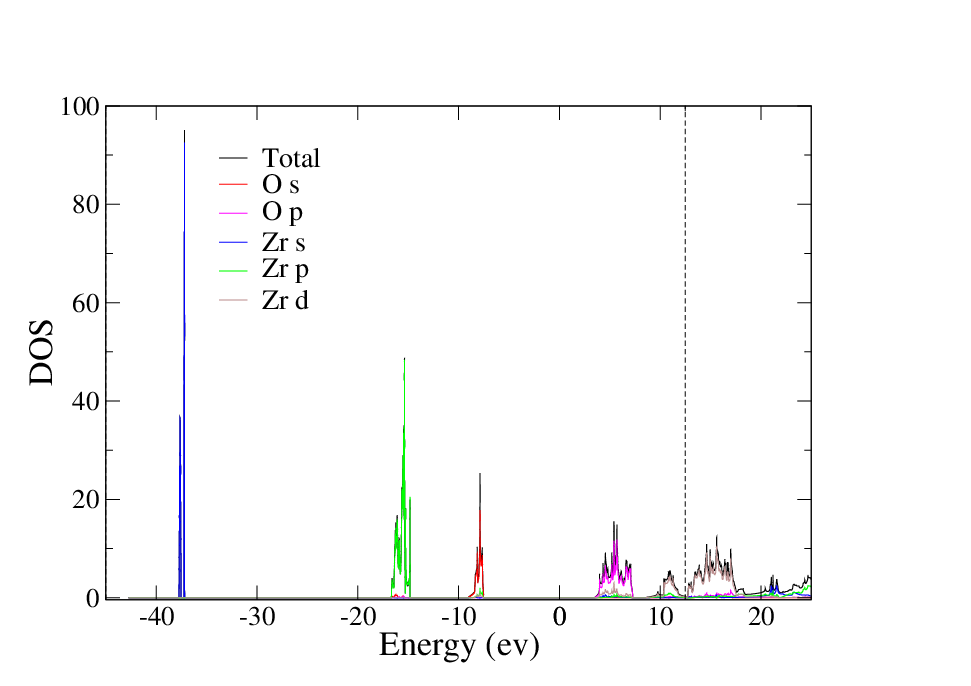


**Figure S12.** Total and site-projected partial DOS for the hexagonal ZrO structure. The Fermi energy is marked by a dashed line.
